# Quality of Physical Activity Participation Among Adults with Disabilities Through Pandemic Restriction

**DOI:** 10.1177/00084174231160954

**Published:** 2023-05-15

**Authors:** Gordon Tao, Gurkaran Singh, Ethan Simpson, Alfiya Battalova, Isabelle Rash, Somayyeh Mohammadi, Julia Schmidt, Jaimie Borisoff, Ben Mortenson, William C. Miller

**Keywords:** Disabled persons (MeSH), Exercise (MeSH), Pandemic, Physical activity, Social participation, Activité physique, exercice (MeSH), pandémie, participation sociale, personnes ayant une incapacité (MeSH)

## Abstract

**Background.** Physical activity (PA) is essential for maintaining well-being in adults with disabilities. This population experienced reduced PA during the COVID-19 pandemic; yet, the impact on quality of PA participation remains unclear. **Purpose.** This secondary analysis explored how pandemic restrictions impacted six experiential dimensions of quality of PA participation among adults with disabilities. **Methods.** An exploratory sequential mixed-methods design, including semi-structured interviews (*n*  =  10) and self-reported surveys (*n*  =  61), was conducted in May-2020 and February-2021. Quality of PA participation was measured using the Measure of Experiential Aspects of Participation (MeEAP). Participants included community-dwelling adults over 19 years of age (mean 59.2  ±  14.0 years) living with stroke, spinal cord injury, or other physical disabilities. **Findings.** Directed content analysis identified three themes related to adjusting PA participation for restrictions, motivation barriers, and valuing social support. These themes highlighted five factors, such as resilience, as potential quantitative predictors of quality of PA participation. While paired correlations with MeEAP scores were observed, these factors were not statistically predictive in multiple regression analysis (adjusted *R*^2^ = −0.14, *F*(10,50)  =  0.92, *p*  =  .53). **Implications.** The interplay between *Meaning*, *Autonomy*, *Engagement*, and *Belongingness* dimensions of quality of PA participation was complex, with an emphasized role for mental health, in adults with disabilities.

## Résumé

**Description.** L’activité physique est essentielle pour préserver le bien-être des adultes qui ont des incapacités. Cette population a dû réduire ses activités physiques pendant la pandémie de COVID-19; toutefois, les conséquences sur la qualité de la participation aux activités physiques restent incertaines. **But.** Cette analyse secondaire explore la manière dont les restrictions liées à la pandémie ont affecté six dimensions expérientielles de la qualité de la participation aux activités physiques chez les adultes ayant des incapacités. **Méthodologie.** Une étude à méthode mixte utilisant un devis séquentiel exploratoire, comprenant des entretiens semi-dirigés (n  =  10) et des enquêtes où les informations étaient autodéclarées (n  =  61), a été réalisée en mai 2020 et février 2021. La qualité de la participation aux activités physiques a été mesurée à l’aide de l’outil Measure of Experiential Aspects of Participation (MeEAP). Les participants étaient des adultes de plus de 19 ans vivant dans la collectivité (moyenne de 59,2  ±  14,0 ans) et composant avec les suites d’un accident vasculaire cérébral, une lésion de la moelle épinière ou un autre handicap physique. **Résultats.** Une analyse descriptive qualitative a permis d’identifier trois thèmes liés à l’ajustement de la participation aux activités physiques en fonction des restrictions, aux obstacles à la motivation et à la valorisation du soutien social. Ces thèmes ont mis en évidence 5 facteurs, tels que la résilience, comme prédicteurs quantitatifs potentiels de la qualité de la participation aux activités physiques. Bien que des corrélations appariées avec les scores MeEAP aient été observées, ces facteurs n’étaient pas statistiquement prédictifs dans l’analyse de régression multiple (R^2^ ajusté  =  -0,14, F[10,50]  =  0,92, p  =  0,53). **Conséquences.** L’interaction entre les dimensions de la qualité de la participation aux activités physiques que sont le *sens*, l’*autonomie*, l’*engagement* et l’*appartenance* était complexe, avec un rôle accentué pour la santé mentale, chez les adultes présentant des incapacités.

## Introduction

The coronavirus 2019 (COVID-19) pandemic prompted sweeping and worldwide restrictions on in-person gatherings, socialization, and services. These restrictions were broadly associated with decreases in mental health and quality of life (Zacher & Rudolph, 2021). Adults with disabilities were particularly impacted as pandemic responses were often not disability-inclusive, for example, disruption or cessation of in-patient rehabilitation services or assistive device programs (Jesus et al., 2021). In their scoping review of disparities experienced by adults with disabilities during the pandemic, Jesus and colleagues highlighted reduced physical activity (PA) leading to health and functional decline such as sarcopenia.

PA involves any body movements driven through energy expenditure by skeletal muscles. PA is an occupation where both quantity and quality play key roles in maintaining physical and mental health, as well as in promoting well-being in adults with disabilities (Martin Ginis et al., 2021; Saebu, 2010). PA for adults with disabilities typically occurs through engagement with prescribed exercise, activities of daily living, and through sports and leisure activities with modifications or adapted equipment (Martin Ginis et al., 2021). The contributions to quantity of PA in adults with disabilities can be understood through the Integrated Model of Physical Activity and Disability (van der Ploeg et al., 2004). This model highlights mobility through the built world, social influences such as family and caregivers, and environmental factors such as transportation and accessibility, as well as personal factors, including health condition, psychological factors such as self-efficacy, and demographic factors such as age and sex that influence PA behaviour. Restrictions during the early pandemic impacted many of these factors and resulted in decreased PA (Caputo & Reichert, 2020). Sedentary behaviour often leads to secondary health problems among adults with disabilities such as cardiovascular disease or diabetes; participation in PA helps reduce or prevent such secondary problems through improved health outcomes and by reducing physical barriers (Martin, 2013; Richardson et al., 2017). Moreover, such secondary health problems can also be risk factors for increased severity of COVID-19 outcomes (Wolff et al., 2021).

Quality of participation encompasses important experiential dimensions of occupation, yet as a construct it remains under-researched and has only recently gained broader attention (Martin Ginis et al., 2017). Quality of participation shares parallels to the concept of Occupational Satisfaction, which relies on feeling a sense of agency, meaningful challenge, and value (Morgan, 2010). Quality of participation can be described by six experiential dimensions of participation (Martin Ginis et al., 2017): *Autonomy* (having independence, choice, or control), *Belongingness* (sense of acceptance, belonging, and respect), *Challenge* (appropriate level of challenge), *Engagement* (feeling motivated, focused, involved), *Mastery* (experiencing achievement, competence, or self-efficacy), and *Meaning* (contributing toward a meaningful goal or feeling social responsibility). These dimensions were determined through a configurative review of participation literature in order to address a gap in how participation was often operationalized primarily in terms of performance of roles or activities (Martin Ginis et al., 2017). The six dimensions were theoretically situated in alignment primarily with self-determination theory and the theory of purposeful work behaviour (Martin Ginis et al., 2017). Moreover, this framework was subsequently operationalized into the Measure of Experiential Aspects of Participation (MeEAP) to measure quality of participation (Caron et al., 2019). As such, this conceptualization serves as a useful theoretical framework for understanding the supports for and detriments of quality of participation in PA. Two programing conditions have been highlighted that support quality PA participation for adults with disabilities: group-based programming with peers and leadership from knowledgeable instructors (Shirazipour et al., 2020). However, reductions and changes to the nature of PA program conditions as a result of COVID-19 pandemic restrictions may impact quality of PA participation for adults with disabilities.

Travel restrictions and facility closures, including fitness and community centres, during pandemic restrictions have directly led to decreases in people's overall ability to move beyond their homes into their surrounding region (Rantanen et al., 2021; Saraiva et al., 2021), and therefore limit their access to PA programs. Moreover, physical distancing requirements also limited the degree to which adults with disabilities may engage in PA socially or with the help of family and caregivers (Jesus et al., 2021). Consequently, adults with disabilities needed to find new approaches to engaging in PA, such as online fitness classes, videos, fitness applications, or telehealth interventions (Brown et al., 2021; Parker et al., 2021). However, it remains unclear how these limitations and adaptations in PA for adults with disabilities may have impacted their qualitative experience of PA, that is, the quality of their PA participation.

Supporting quality of PA participation of adults with disabilities may be particularly important during periods of adversity including social restrictions and limited access to health facilities (Jesus et al., 2021). Understanding how such restrictions impact quality of participation and the ways in which adults with disabilities respond may help to inform future implementation of accessible PA activities during adverse conditions. Therefore, the primary objective of this study was to explore qualitatively the impact of pandemic restrictions on quality of PA participation and factors influencing it among adults with disabilities during the first year of COVID-19 pandemic restrictions. The secondary objective was to determine quantitatively which factors (identified through qualitative exploration) early in the pandemic were predictive of quality of PA participation later in the first year of the pandemic.

## Methods

### Study Design

This secondary analysis study followed an exploratory sequential mixed-methods design (Creswell et al., 2011), including semi-structured interviews and surveys. While data were collected concurrently, data analysis was sequential, with qualitative findings informing quantitative analysis. Data were collected as part of a larger longitudinal study exploring experiences of COVID-19 pandemic restrictions in British Columbia, Canada across a range of populations (Reid et al., 2021). These data included both interview transcripts and survey responses from the same participants. Ethical approval was received from the University of British Columbia and Vancouver Coastal Health (H14-01737). We reported this study according to the Good Reporting of A Mixed Methods Study guidelines (O’Cathain et al., 2008) and the Standards for Reporting Qualitative Research guidelines (O’Brien et al., 2016).

### Participants

To explore a diverse cross-section of how adults with disabilities experienced quality of PA participation, we included community dwelling adults over 19 years of age who self-identified with one of three population groups: people who have experienced stroke, spinal cord injury, or other disabilities. Disabilities may have been child onset or acquired later in life. Participants needed to be comfortable writing and speaking in English and have internet or telephone access. Individuals with moderate to severe cognitive impairment or aphasia were excluded.

Participants were recruited through online postings on research portals, social media of researchers involved in the study, and through a database of past participants with consent to contact. Participants provided informed consent at each point of data collection. Participants were anonymized with a unique ID spanning both their qualitative and quantitative data.

### Semi-Structured Interviews

From the original study (Reid et al., 2021), we purposefully subsampled 10 participants with representation across a range of quality of PA participation and across the three groups of SCI, stroke, and other physical disabilities. Participants were interviewed both at baseline (May-June 2020) and later during the pandemic (December-February 2021), as the first COVID-19 vaccines became available. Interviews were conducted by trained research assistants with academic backgrounds in rehabilitation science or occupational therapy via teleconference (Zoom Video Communications, Santa Clara, USA) for 1–1.5 h. The interview guide (Supplemental 1) included questions related to experiences of daily activities, changes in activities, feelings about these changes, and strategies for coping with pandemic restrictions. The second interview covered the same questions except that it emphasized probing for changes “since the last time.” Participants received a $30 honorarium for each interview.

### Survey

Six self-report measures assessing elements of quality of participation, technology readiness, mental health, social support, and life space mobility were distributed to participants through a secure online data management system (Qualtrics, Provo, USA). Demographic data was also collected via this method, including age, sex, education level, and whether or not participants lived alone. For this study, we included data from all survey respondents who met our inclusion criteria (*n*  =  61) and included predictive factors at baseline (within 1 week before the first interview) and the outcome variable at later in the pandemic (within 1 week before the second interview). Predictive factors were selected based on qualitative findings and the personal and environmental factors highlighted in the Integrated Model of Physical Activity and Disability model (van der Ploeg et al., 2004). All measures collected in the survey may be found in the original study (Reid et al., 2021).

The outcome of interest for this study was quality of PA participation, as measured by the recently developed MeEAP (Caron et al., 2019). The MeEAP is a 12-item measure that assesses six dimensions of quality of participation (*Autonomy, Belongingness, Challenge, Engagement, Mastery,* and *Meaning*) in adults with disabilities (7-point Likert scale; “Strongly Disagree” to “Strongly Agree”). For example, one item for *Belongingness* is “When engaging in physical activity, I feel … accepted by others.” Dimensional scores range from 1 (low quality) to 7 (high quality) and overall quality of participation may be assessed through mean response across all dimensions. In a sample of 228 adults with physical disabilities who participated in employment, mobility, sport, or exercise, the measure had strong model fit (Satorra-Bentler scaled χ^2^(39)  =  58.26, *p* < .001, comparative fit index  =  0.98) and evidenced convergent validity (variance extracted values above 0.5), discriminant validity, and internal reliability (Caron et al., 2019).

To account for shifts in PA participation related to technology usage, we included technology readiness, as a predictive factor measured by the Technology Readiness Index 2.0 (TRI-2). The TRI-2 is a 16-item scale (5-point Likert scale; “Strongly Disagree” to “Strongly Agree”), including subscales for optimism, innovativeness, discomfort, and insecurity which can be scored as an average response (1–5) with higher scores indicating greater readiness. It has evidence of good reliability (Cronbach's α 0.70–0.83) as well as discriminant, convergent, and construct validity (Parasuraman & Colby, 2015).

Factors related to mental health included the total Hospital Anxiety and Depression Scale (HADS-T) score, consisting of the HADS-Anxiety and HADS-Depression subscales (Zigmond & Snaith, 1983). There are 14 4-point Likert scale items scored from 0 to 3 (“none” to “extreme”) for a total maximum HADS-T of 42; higher scores indicate greater anxiety and depression. Mean Cronbach α was observed at 0.83 in patients and the general population (Bjelland et al., 2002).

The Connor-Davidson Resilience Scale 25 (CD-RISC-25) was also used to measure resilience (Connor & Davidson, 2003). This 25-item scale (5-point Likert scale; “Not true at all” to “Nearly all the time”) assesses how well people cope with and recover from stressful events and tragedies. Scored from 0 to 100, higher scores indicate greater resilience. The reported Cronbach α for this measure was 0.93 in a general population (Connor & Davidson, 2003).

The Multidimensional Scale of Perceived Social Support (MSPSS) was used to measure social support (Zimet et al., 1988). This 12-item scale (7-point Likert scale; “Strongly Disagree” to “Strongly Agree”) assesses support across subscales of family, friends, and significant others with a score range of 12–84; higher score indicates higher perceived support. In adolescents and adults, the Cronbach α was observed to be 0.88 (Zimet et al., 1988).

To account for mobility changes due to pandemic restrictions, the Life Space Assessment (LSA) was used to measure life space mobility based on the past 4 weeks: the extent of space through which individuals move (room, home, or region), the degree of independence, and frequency of movement (Baker et al., 2003). Each parameter is scored from 0 to 5 and the composite score ranges from 0 to 120 where higher scores indicate greater life space mobility. The test-retest intraclass correlation coefficient has been observed to be 0.88 across 9 days in people living with SCI (Lanzino et al., 2016).

### Analyses

We summarized survey responses with descriptive statistics including mean and standard deviation using IBM SPSS Statistics 25 (IBM, Armonk, USA). Missing data (35 of 6205 item responses across measurement scales) were replaced using multiple imputation.

To address the primary objective, we selected a subsample of interviews from 10 participants out of all the survey respondents and who represented the range of MeEAP scores from 1 to 7 across the three disability groups. Interviews were transcribed verbatim. We used directed content analysis with a constructivist approach to explore pandemic impacts on quality of PA participation within the interviews at both baseline and later-pandemic (Hsieh & Shannon, 2005). GS and ES performed the analysis, using the six experiential dimensions of participation (Martin Ginis et al., 2017) as direction. All transcripts were read repeatedly to achieve immersion and sections related to PA participation were highlighted. Highlighted sections were then coded according to the six experiential dimensions of participation where possible. Codes were gradually incorporated into sub-groups and ultimately themes (Hsieh & Shannon, 2005). Qualitative analyses were performed using NVivo 12. Trustworthiness strategies included investigator triangulation and peer debriefing with co-authors (Nowell et al., 2017). GS and ES have academic backgrounds in mobile health apps for individuals living with SCI and in kinesiology related to improving wellbeing and athletic performance, respectively. Both have experience with qualitative analysis, supporting a diverse and collaborative analysis.

To address the secondary objective, we used qualitative findings to guide our quantitative analysis (Creswell et al., 2011). Integrating qualitative and quantitative methods, we identified potential predictors of quality of PA participation within the identified themes, informed by the six experiential dimensions of participation, and selected corresponding measures from the survey. This process involved discussion amongst the research team, comparing and drawing connections between the emerging themes, survey, research question, and Integrated Model of Physical Activity and Disability. To explore bivariate associations, we constructed a correlation matrix between potential predictors and MeEAP scores for each dimension. We interpreted correlation strength according to *r*: 0 ≤ negligible < 0.3 ≤ weak < 0.5 ≤ moderate < 0.7 ≤ high < 0.9 ≤ very high ≤ 1.0 (Mukaka, 2012). We used multiple regression to examine the predictive relationship between potential predictors, and mean MeEAP. All quantitative analyses were conducted in SPSS 25 with α  =  0.05.

## Findings

### Participant Description

Survey data from 61 participants, including 26 who have experienced stroke, 22 with SCI, and 13 with other disability (e.g., mental health disabilities, amputation, cerebral palsy, brain injury, etc.), from the original study were included in the quantitative analysis. Table 1 summarizes demographic proportions and measures. Participants’ ages ranged from 30 to 82 years. The majority of participants had completed post-secondary education, including college, university, graduate, and post-graduate degrees. Most participants (*n*  =  38) lived with others. Some participants (*n*  =  6) did not disclose a category of disability, but described mobility limitations or limitations related to chronic health conditions such as cancer, diabetes, or seizures post-stroke.

**Table 1:** Sample (*n*  =  61) Summary Including Demographics, Predictors, and MeEAP Scores

Variables	Measure range	Mean (SD)
**Age**		59.2 (14.0)
30–49		*n* = 13
50–64		*n* = 22
65–79		*n* = 24
80 +		*n* = 2
**Male**		59%
**Post-secondary education**		92%
**Living alone**		38%
**Disclosed disability**		
Physical disability		*n* = 40
Mental health disability		*n* = 5
Sensory disability		*n* = 5
Cognitive disability		*n* = 3
Learning disability		*n* = 2
**Technology readiness (TRI-2)^+^**	1–5	3.3 (0.59)
**Anxiety (HADS)^−^**	0–21	5.2 (4.2)
**Depression (HADS)^−^**	0–21	4.6 (3.5)
**Resilience (CD-RISC-25)^+^**	0–100	71.5 (14.4)
**Social support (MSPSS)^+^**	1–7	5.2 (0.18)
**Life space assessment (LSA)^+^**	0–120	50.2 (23.1)
**MeEAP^+^**	**Measure range**	**Mean (SD)**
Autonomy	1–7	5.6 (1.3)
Belongingness	1–7	5.4 (1.2)
Challenge	1–7	5.3 (1.1)
Engagement	1–7	5.2 (1.4)
Mastery	1–7	5.2 (1.2)
Meaning	1–7	5.4 (1.0)
Overall mean	1–7	5.3 (1.0)

Variables with (^+^) indicate higher is better and (^−^) indicates higher is worse.

Our purposive subsampling of participant interviews for directed content analysis included three participants from the stroke group (STR), four from SCI, and three from other disability (DIS). Their MeEAP scores ranged from 2.5 to 6.9 (Table 2). The subsample included four female and six male participants with an mean age of 61.8 years.

**Table 2:** Individual Demographics and Measures for Sub-sample (*n*  =  10) of Semi-Structured Interviews

	STR1	STR 2	STR 3	SCI 1	SCI 2	SCI 3	SCI 4	DIS 1	DIS 2	DIS 3
**Age**	81	31	64	53	62	48	71	79	58	71
**Sex**	M	F	F	F	F	M	M	M	M	M
**MeEAP^+^**										
Autonomy	6.0	7.0	6.0	4.0	6.0	1.0	6.5	7.0	7.0	7.0
Belongingness	6.0	7.0	3.5	4.0	6.0	2.5	7.0	5.5	7.0	4.0
Challenge	5.0	7.0	4.5	3.5	6.0	3.0	6.0	7.0	5.5	5.0
Engagement	6.0	7.0	2.0	3.5	5.5	3.0	6.5	7.0	5.5	3.0
Mastery	6.0	7.0	4.0	3.5	6.0	1.5	6.5	6.0	6.0	5.0
Meaning	6.0	6.5	3.5	3.5	5.5	4.0	6.0	5.5	4.5	4.0
Mean score	5.8	6.9	3.9	3.7	5.8	2.5	6.4	6.3	5.9	4.7
**TRI 2.0^+^**	3.3	3.1	3.0	2.9	2.7	4.3	4.9	3.7	3.0	2.3
**HADS-T^−^**	10	4	5	20	37	8	2	4	17	13
**CD-RISC-25^+^**	73	66	67	75	30	88	91	78	67	53
**MSPSS^+^**	6.7	5.8	6.3	5.9	4	5.5	6.7	6.8	5.8	1.4
**LSA^+^**	53	90	76	37	18	66	46	32	50	72

Variables with (^+^) indicate higher is better and (^−^) indicates higher is worse.

TRI 2.0  =  Technology Readiness Index 2.0; HADS-T  =  Hospital Anxiety and Depression Scale Total; CD-RISC-25  =  Connor-Davidson Resilience Scale 25; MSPSS  =  Multidimensional Scale of Perceived Social Support; LSA  =  Life Space Assessment.

### Qualitative Findings

All 10 participants included in the sub-sample had completed initial and follow-up interviews in the original study. Therefore, a total of 20 interviews were included in the analysis. Three main themes addressing how quality of PA participation was impacted by pandemic restrictions were identified, including: (1) adjusting PA participation to meet restriction requirements, (2) coping with barriers in motivation for PA participation, and (3) valuing social support in PA participation (Table 3).

**Table 3:** List of Themes and Sub-themes

Theme	Sub-theme
Adjusting PA participation to meet restriction requirements	Experiencing limitations in PA participation
	Taking initiative to stay physically engaged
Coping with barriers in motivation for PA participation	External barriers decreased opportunity and motivation for PA participation
	Internal factors linked to decreased motivation for PA participation
Valuing social support in PA participation	Valuing structural social support
	Valuing personal and communal social support

### Adjusting PA Participation to Meet Restriction Requirements

Participants described how the COVID-19 pandemic necessitated adjustments in their PA participation. The first sub-theme explored how participants experienced limitations in PA participation. Participants highlighted how they increasingly engaged in sedentary activities, such as watching TV, engaging in arts and crafts, or being on the computer in response to restrictions. For example, one individual living with stroke (STR-3, Male, Age 71) stated:

Oh, I was tired of being inside. […] I just felt like I want to go to a movie. I want to get together with friends and it's not as easy. Well, I watch movies at home.

Participants also described how this shift towards sedentary activities could be felt in the workplace settings, where videoconferencing has supplanted physically commuting to work. For example, one individual living with stroke (STR-2, Male, Age 58) described how the pandemic has resulted in shifting their entire workplace onto Zoom:

I'm doing new things about technology for my own work. I am already familiar with Zoom [video conferencing] and whatnot [and becoming] more familiar with change tools for online teaching. […] So I've learned new skills and redesigning business models, and we created a new online system.

Conversely, participants also highlighted how technology-based activities typically perceived as sedentary could still be used to promote *Meaningful* PA participation. One participant living with stroke (STR-3, Male, Age 71) described how they participated in exercise programs over YouTube and Zoom videoconferencing:

I […] came across YouTube with a lot of [exercises even for seniors]. I might take advantage of that, and then use my computer, plug it into my TV, and have my wife and I daily do some simple exercises for seniors. Move around, doing leg exercises, that sort of thing. So utilize what we have with the high tech.

Participants also highlighted limitations in *Autonomy* such as going out much less frequently due to pandemic-related lockdowns and to avoid becoming severely sick while already living with a chronic health condition. One participant living with SCI (SCI-2, Female, Age 62) stated:

Initially, the first two months all the stores were closed, all the restaurants and cafes, and bars. They were all closed so it was very restrictive. And I mean, yes, the grocery stores were open, but my wife was like ‘you’re not going anywhere near the grocery stores, stay at home. I don't want you getting infected or anything like that in the grocery store.’ So she did all the grocery shopping.

Coping with restrictions, particularly at baseline, participants seemed to demonstrate resilience by engaging in non-traditional exercise activities (e.g., household chores) that provided sufficient *Challenge* as part of their PA participation as a substitute to traditional exercise such as walking, going for “a short run for 10 to 20 minutes” (SCI-3, Male, Age 48), or going to the gym.

The second sub-theme explored ways that participants experienced *Autonomy* by taking initiative to participate in PA since the start of the pandemic. This often involved developing self-led home workouts, including one participant with an SCI who stated:

*Yeah, I have some equipment here and some hand weights. And […] I use that to great effect. I […] used to be a former, real intense athlete, And I think […] I just use those lessons that I learned before to help me in this time as well.*—SCI-4, Male, Age 71

Similarly, a key component of exercise included stretching, which participants also found ways to do from home:

*I get up in the morning and I do a series of stretching exercises in my bed before I get up. I go into the bathroom and I have a real set up, and I do some more stretchers there. […] I then go out on the steps […] outside our apartment, which have a flight of stairs going up. […] I do more stretching exercises and other exercises there.*—DIS-1, Male, Age 81

As previous PA options outside of the home became limited, alternative PA strategies such as wheeling became more important:

*I get in my manual wheelchair and I go exercising for about an hour. Right outside here of our condo. We have pedestrianized a couple of blocks going all uphill one way and fortunately downhill the other way. So I just do a couple of loops around here for now and get some exercise.*—SCI-2, Female, Age 62

Participants suggested PA was important for health and a means of distraction during times of COVID-19 restriction and anxiety:

*If I exercise a lot, my body tends to move around a little bit […] It's a little harder to distract yourself sometimes from the time of COVID-19 […] so that's what keeps me from not going crazy by staying busy. Staying busy keeps me not thinking about the pain I’m in and I think that's maybe a little bit of a challenge sometimes.*—SCI-4, Male, Age 71

Similarly, simple activities like household chores were sometimes used as a means to stay active while also passing some time.

### Coping With Barriers in Motivation for PA Participation

Participants highlighted how both environmental factors and mental health during the COVID-19 pandemic impacted motivation for PA participation. For the first sub-theme, there were two key environmental factors that impacted participant's motivation for PA participation: closures and weather. The closure or cancellation of several facilities and programs such as support groups restricted participants’ *Autonomy* as their PA choices were curtailed as well as limitations in *Belongingness*:

*Well, unfortunately, we [were forced to] quit many activities. Oh, you know, […] they shut everything down. So we go to our local community club. Well, that was closed. So we haven't been meeting since that time. And there has been talk that perhaps we won't meet again till next year. This is still to be determined.*—STR-3, Male, Age 71

Participants also emphasized how the closures of gyms still impacted their frequency of PA participation. For example, one individual with SCI stated:

*You're limited to basically going outside once a day as opposed to two or three times a day. […] So I can't go to gym class [as frequently].*—SCI-1, Female, Age 53

Responding to indoor restrictions, participants also highlighted how influential weather became on *Autonomy* and *Engagement* in PA participation, with participants more likely to find PA engaging during nice weather:

*If the weather is good, I just walk around outside. […] Just walk around the compound. […] But I this one week I did not walk because it's raining, so I don't feel like going out. So I just stay home.*—SCI-3, Male, Age 48

Some participants found alternative options and adapted the challenge of these PAs to suit them, even when the weather was bad. For example, one individual living with SCI described how “if it's not raining, [they] will go out on [their] manual wheelchair to get some exercise but if it's raining, [they] still need to exercise, so [they] go in the parking base in the parking lot downstairs in [their] condo block and do a couple of rounds in the [parking] basement garage” (SCI-2, Female, Age 62).

For the second sub-theme, internal factors that decreased motivation for PA participation (i.e., reduced *Autonomy* and *Engagement*) included fatigue and mental health considerations such as anxiety and depression. In terms of fatigue, the pandemic resulted in some participants experiencing less motivation to do activities of daily living. One individual living with SCI stated:

*In almost every way, in almost everything, like daily living, I don't feel like moving. I don't feel like doing anything. Plus, nowadays, like wintertime we still get some activities, but now I think my activities are getting lesser and lesser.*—SCI-3, Male, Age 48

Participants also emphasized how living with depression played a major role in them not wanting to get out of bed and was also accompanied by mood swings:

*Some days I'm very depressed. I don't want to get out of bed. Other days, I'm okay. My default mood seems to be low grade depression. That's just how it is.*—DIS-3, Female, Age 64

Similarly, participants described how anxiety also influenced motivation for PA participation due to “concerns about catching the disease,” and therefore felt that going out “would be a very dangerous thing to do” (DIS-1, Male, Age 81).

## Valuing Social Support in PA Participation

Participants emphasized the value of receiving social support in PA participation. For the first sub-theme, participants highlighted how they benefited from structural social support. Participants emphasized how they participated in structured classes or sessions with physiotherapists, fitness instructors, etc., to improve *Engagement* and *Belongingness* dimensions of PA participation:

*I was going to Pilates twice a week, and it closed until yesterday. So for 2.5 months, I didn't go to something that I really enjoyed a lot. Yesterday was my first day back, and I feel much more motivated now, to take better care of myself.*—DIS-3, Female, Age 64

However, at baseline, participants discussed how these structured programs were interrupted, negatively impacting their health and well-being. For example, SCI-4, Male, Age 71 stated:

*Well, I think the biggest thing that I could speak of now for myself, as I have had 3 to 4 physiotherapists sessions a week for five years and starting in I think, the 21st of March [2020] that ended up until June 1^st^ [2020]. And that was a massive interruption in my life. It was a massive uptick in pain and discomfort, and it was really the most challenging part of this whole ordeal for myself.*

The challenges of interruptions in structured PA programs were not highlighted by participants at baseline.

For the second sub-theme, participants also emphasized the value of receiving support in PA participation from family, friends, and their local communities:

*So it's my wife's job, really, to do the housework, and also to monitor me, keep me in good shape, push me around when she needs to* –DIS-1, Male, Age 81

However, while one participant highlighted how they would feel better about participating in PA if they “got together with somebody and stick to a routine,” they felt the COVID-19 pandemic situation prevented this (STR-1, Male, Age 81). Furthermore, participants also felt that being accommodated by others for their unique disability-related needs played a significant role in improving *Belongingness* in PA participation. For example, participants appreciated that individuals riding bikes and strollers would “let them pass on sidewalks” (SCI-1, Female, Age 53) to promote accessibility while abiding by social distance guidelines.

### Quantitative Predictors

Based on the qualitative findings, we identified five factors from the survey as potential predictors of quality of PA participation. For example, the authors discussed how resilience, as measured on the original survey, resonated with how participants described the ways in which they coped with restrictions by taking initiative to stay physically engaged; this construct was also consistent with psychological factors predicting PA behaviour in the Integrated Model of Physical Activity and Disability. From the survey data, we used the measures TRI-2 to address (1) new technology usage, HADS to address (2) mental health, CD-RISC-25 to address (3) resilience, MSPSS to address (4) social support, and LSA to address (5) mobility limitations due to pandemic restrictions (Table 1).

At baseline, technology readiness was on average near the midpoint (TRI-2  =  3) of the scale, indicating neutral attitude. Both anxiety and depression were on average within the normal range (HADS < 8), with 31.1% and 19.7% of participants respectively falling in the “borderline abnormal” or “abnormal” range. Resilience ranged from 30 to 100. On average, social support in our sample was high (MSPSS ≥ 5.1). Life space mobility ranged from 6, indicating little mobility beyond one's room, to 120, indicating regular mobility beyond one's town without assistance.

At later pandemic, participants somewhat agreed with experiencing quality participation in PA (5 ≤ MeEAP < 6) overall. Scores were also similar across the 6 MeEAP dimensions. The lowest individual subscale score appeared in Autonomy (MeEAP-*Autonomy*  =  1).

Exploring the six dimensions of quality of PA participation measured by the MeEAP, the correlation matrix (Supplemental 2) showed weak associations between MeEAP-*Belongingness* and HADS-Anxiety (*r*  =  −.35, *p* < .01); MeEAP-*Challenge* and HADS-Anxiety (*r*  =  −.36, *p* < .01), MeEAP-*Mastery* and HADS-Anxiety (*r*  =  −.26, *p* < .05); MeEAP-*Meaning* and TRI-2 (*r*  =  .31, *p* < .05) as well as resilience measured by CD-RISC-25 (*r*  =  .33, *p* < .01). Mean MeEAP also showed weak association with HADS-Anxiety (*r*  =  −.29, *p* < .05)*.*We included in our model demographics (age, sex, education level, living alone, and group), TRI-2 (technology readiness), total HADS score (anxiety and depression), CD-RISC-25 (resilience), MSPSS (social support), and LSA (life-space mobility). Collinearity diagnostics showed all VIF < 2. Our multiple regression model was not statistically significant (adjusted *R*^2^  =  −0.14, *F*(10,50)  =  0.92, *p*  =  .53), indicating no explanatory relationship between independent variables and variance of mean MeEAP scores.

## Discussion

From interviews conducted early in the pandemic and 8–10 months later, three themes emerged that delineated the impacts of COVID-19 pandemic restrictions on quality of PA participation in adults with disabilities. Participants highlighted how they adjusted PA participation, how motivation changes influenced quality of PA participation, and the role of social support in creating quality participation. Within these themes, we identified new technology usage, mental health, resilience, social support, and life-space mobility as potential predictors of quality of PA participation 10 months later. However, while these predictors saw weak associations with MeEAP dimensions in terms of individual correlations, the multiple regression ultimately saw no statistically significant relationship between these predictors and overall quality of PA participation as measured by mean MeEAP. This lack of alignment between qualitative and quantitative data suggests that the relationship between quality of PA participation later in the pandemic and how participants presented at the outset of the pandemic was more complex than expected.

In the theme “Adjusting PA participation to meet pandemic restrictions,” participants described how the pandemic-imposed circumstances limited their typical PA engagement and greatly increased their sedentary time. Participants reported how they found new ways to meaningfully engage in PA in their home, both through technology (e.g., Zoom classes, YouTube tutorials) and by adapting PA to involve home-oriented activities. These experiences are consistent with pandemic experiences of general population adults who struggled with gym closures and re-engaged with PA through technology such as smart-watches and online videos (Petersen et al., 2021). This capacity to readily adjust to online PA may also reflect the weak correlation we observed between technology readiness at the outset of restrictions and the *Meaning* dimension of PA participation 10 months later. Contextually, we may consider that PA may have taken on new meanings, that is, contributing towards personal and socially meaningful goals for feeling a sense of responsibility to others, during pandemic restrictions. During the early pandemic, there was greater health-information seeking as well as emphasis placed on adhering to health guidelines (Roberts et al., 2021). For example, British Columbia health guidelines emphasized the importance of PA during the pandemic to help reduce severity of COVID-19 infection and to support mental health as well as doing so in ways that contribute to safety of the community (Ministry of Health, 2022). Such considerations may contribute to the meaningfulness of engaging in PA through technology.

While participants described negative impacts to the *Autonomy* dimension of PA participation (i.e., having independence, choice, and control) in terms of reduced life-space mobility, for example, going out much less frequently, this association was not reflected in the quantitative analysis. Perhaps participants’ subsequent adjustments in PA to restrictions mitigated this association by creating new options to support *Autonomy*. Similarly, participants substituted PA goals typically accomplished outside of the home for familiar ones within and around the home that they found sufficiently *Challenging* (e.g., chores or going around the block) and as a means of distraction during pandemic restrictions. Indeed, adults with disabilities are typically familiar with the task of adapting to mobility limitations and barriers in the external environment (Rimmer et al., 2004) as well as adapting their home environment for their occupations (Lund & Nygard, 2004). As such, past experiences of adults with disabilities with adaptation may aid them in developing quality PA participation at home, provided appropriate guidance in adapting PA goals and access to technology. Indeed, individualized home-based PA interventions for adults with disabilities are well researched (Ma & Martin Ginis, 2018). The importance of making such interventions more broadly accessible to adults with disabilities has been underscored by pandemic restrictions, as having more such choices would at least support quality of PA participation in terms of *Autonomy*.

In “Coping with barriers in motivation for PA participation,” participants described how both circumstances outside their control and their psychological reactions to pandemic restrictions reduced the *Engagement* dimension of their PA participation in terms of motivation. In this context, the *Engagement* and *Autonomy* dimensions of participation appear interlinked, as some participants felt less in control over the PA they did have available to them, that is, environmental barriers such as poor weather reducing motivation to exercise outside. Pandemic-related depression and anxiety were described as powerful challenges to mental health, and additional barriers that reduced motivation for participants to take part in PA. This may be reflected by the weak correlation we observed between the *Challenge* dimension of participation and anxiety, whereby mental distress creates the perception that available PA is too difficult or risky. This explanation would align with general conceptions of self-efficacy whereby belief of control over exercise is associated with more exercise participation (Neupert et al., 2009). Likewise, anxiety has been shown to influence other aspects of *Engagement*, such as focus or experiencing flow state (Jackson et al., 1998). Wolf and colleagues’ review further highlighted this association between PA quantity and mental health during the COVID-19 pandemic across 21 studies and 5 continents (Wolf et al., 2021). Our findings expand on this relationship through experiences that elucidate how different dimensions of PA participation quality interrelate with mental health, individual perceptions, and environmental circumstance for adults with disabilities during adversity.

In “Receiving social support in PA participation,” participants highlighted the importance of structural and community support for quality PA participation. Structured PA sessions with experts and peers were particularly important as their absence resulted in experiences of occupational deprivation, while the restoration of such activities appeared to support *Engagement* and *Belongingness* dimensions of participation. This is consistent with established literature demonstrating the benefits of group PA and its association with social connectedness and empowerment (Farrance et al., 2016; Harden et al., 2015). Quality participation was also experienced through community support enhancing *Meaning* through accountability to others and *Belongingness* in the form of social acceptance of participants’ visible disability status. Moreover, perceived social support is known to mitigate negative psychological conditions such as anxiety (Roohafza et al., 2014). As such, perhaps it was social support in the early pandemic, which may explain the weak negative correlation observed between HADS-Anxiety and MeEAP-*Belongingness*. Social support may be particularly important during transitionary periods such as the early pandemic as social relationships can be crucial for supporting the initiation of adapted PA (Javorina et al., 2020).

Despite observing individual correlations between quality of participation dimensions and potential predictors, overall quality of PA participation was not predicted by any factors at baseline. This finding contrasts with previous studies that identified quantitative relationships between gender and quality of participation as measured by the MeEAP in context of leisure-time PA for adults with disabilities (Koch et al., 2022). Moreover, another study showed MeEAP scores as a moderator in the relationship between PA and mental health in terms of loneliness (Santino et al., 2021). In contrast to our regression results, our qualitative findings were consistent with other evidence describing barriers and facilitators of quality PA participation (Fakolade et al., 2021; Fong et al., 2021). With this consideration, the changes experienced by our participants through adverse restrictions and their diverse adaptations to them, for example, using online tools to compensate for facility closures, may have complexified the ways in which quality PA participation was both supported (e.g., with social support) and hindered (e.g., by mental health challenges) such that prediction of a single score became untenable.

### Limitations

The MeEAP remains a relatively new measurement tool and its applications for measuring quality of participation are still being explored. Our findings suggest that, in context of our prognostic examination of potential predictors, quality of PA participation may be better explored using the MeEAP according to each individual dimension rather than as a composite measure. Notably, the mean score across each dimension was quite similar, ranging from 5.2 to 5.6 (between “Agree” and “Somewhat Agree”) in our sample. This is consistent with other samples involving the MeEAP involving adults with disabilities who observed high mean scores across each dimension (Bremer et al., 2022; Santino et al., 2021). Therefore, future research may wish to use the MeEAP to examine circumstances of lower quality participation.

Considering predictor measures, our predictor selections were limited to the original survey and interviews, which were not designed specifically to assess relationships with quality of PA participation at baseline. Moreover, the survey sample was relatively small, limiting the ability to detect more nuanced regression relationships. However, the original study was intended to explore the overall impact of pandemic restrictions on daily activities, including PA.

Given that pandemic restrictions involved regional and temporal differences across Canada and internationally, generalizability was limited by our focus on British Columbia, Canada, where curfews and more severe lockdown measures were not implemented (Ritchie et al., 2020). Moreover, ours was a sample of convenience; participants were resourced well enough to have reliable teleconference capability and time for research participation. As such we may not have captured experiences of more severe adversity, for example, greater psychological adversity or homelessness. However, commonalities remain in terms of coping with facility closures and social distancing.

## Conclusion

In this study, we have advanced the understanding of the experiential dimensions of PA participation experienced by adults with disabilities during the adverse condition of COVID-19 pandemic restrictions. Our findings emphasized the complex and diverse interplay between *Meaning, Autonomy*, *Engagement*, and *Belongingness* in PA participation. While many experiences were similar to that of general populations (e.g., finding new *Meaning* by adapting PA participation and relying on social support), the unique context of disabilities also manifested in the urgency of participants’ anxiety over contracting COVID-19 while living with disability and over loss of access to resources for adults with disabilities such as support groups that facilitate quality participation in PA. Our findings also indicated weak bivariate associations between quality of PA participation and anxiety, resilience, and technology readiness. However, more robust modeling of quality participation determinants that reflect such relationships have yet to be observed. Quality of participation is a construct that has only recently been structurally characterized (Martin Ginis et al., 2017) and the appropriate application of the MeEAP as its quantitative measure may require more elucidation. Future work may apply our findings by exploring how home-based PA interventions for adults with disabilities may better support quality participation*.*

## Supplemental Material

sj-docx-1-cjo-10.1177_00084174231160954 - Supplemental material for Quality of Physical Activity Participation Among Adults with Disabilities Through Pandemic Restriction Click here for additional data file. Supplemental material, sj-docx-1-cjo-10.1177_00084174231160954 for Quality of Physical Activity Participation Among Adults with Disabilities Through Pandemic Restriction by Gordon Tao, Gurkaran Singh, Ethan Simpson, Alfiya Battalova, Isabelle Rash, Somayyeh Mohammadi, Julia Schmidt, Jaimie Borisoff, Ben Mortenson and William C. Miller in Canadian Journal of Occupational Therapy

sj-docx-2-cjo-10.1177_00084174231160954 - Supplemental material for Quality of Physical Activity Participation Among Adults with Disabilities Through Pandemic Restriction Click here for additional data file. Supplemental material, sj-docx-2-cjo-10.1177_00084174231160954 for Quality of Physical Activity Participation Among Adults with Disabilities Through Pandemic Restriction by Gordon Tao, Gurkaran Singh, Ethan Simpson, Alfiya Battalova, Isabelle Rash, Somayyeh Mohammadi, Julia Schmidt, Jaimie Borisoff, Ben Mortenson and William C. Miller in Canadian Journal of Occupational Therapy

## References

[bibr1-00084174231160954] Baker P. S. Bodner E. V. Allman R. M. (2003). Measuring life-space mobility in community-dwelling older adults. Journal of the American Geriatrics Society, 51(11), 1610–1614. 10.1046/j.1532-5415.2003.51512.x 14687391

[bibr2-00084174231160954] Bjelland I. Dahl A. A. Haug T. T. Neckelmann D. (2002). The validity of the hospital anxiety and depression scale: an updated literature review. Journal of Psychosomatic Research, 52(2), 69–77. 10.1016/S0022-3999(01)00296-3 11832252

[bibr3-00084174231160954] Bremer E. Liska T. M. Arbour-Nicitopoulos K. P. Best K. L. Sweet S. N. (2022). Examining long-term motivational and behavioral outcomes of two physical activity interventions. The Journal of Spinal Cord Medicine, 0(0), 1–11. 10.1080/10790268.2022.2033935 PMC10446818 35254230

[bibr4-00084174231160954] Brown M. O’Connor D. Murphy C. McClean M. McMeekin A. Prue G. (2021). Impact of COVID-19 on an established physical activity and behaviour change support programme for cancer survivors: an exploratory survey of the Macmillan move more service for Northern Ireland. Supportive Care in Cancer, 29(10), 6135–6143. 10.1007/s00520-021-06165-1 33811517 PMC8019085

[bibr5-00084174231160954] Caputo E. L. Reichert F. F. (2020). Studies of physical activity and COVID-19 during the pandemic: a scoping review. Journal of Physical Activity and Health, 17(12), 1275–1284. 10.1123/jpah.2020-0406 33152693

[bibr6-00084174231160954] Caron J. G. Martin Ginis K. A. Rocchi M. Sweet S. N. (2019). Development of the measure of experiential aspects of participation for people with physical disabilities. Archives of Physical Medicine and Rehabilitation, 100(1), 67–77.e2. 10.1016/j.apmr.2018.08.183 30268805

[bibr7-00084174231160954] Connor K. M. Davidson J. R. T. (2003). Development of a new resilience scale: the Connor-Davidson resilience scale (CD-RISC). Depression and Anxiety, 18(2), 76–82. 10.1002/da.10113 12964174

[bibr8-00084174231160954] Creswell J. Klassen A. C. Plano V. Smith K. C. (2011). Best practices for mixed methods research in the health sciences. National Institutes of Health. https://obssr.od.nih.gov/sites/obssr/files/Best_Practices_for_Mixed_Methods_Research.pdf

[bibr9-00084174231160954] Fakolade A. O. Latimer-Cheung A. E. Shirazipour C. H. (2021). Quality participation: perspectives of physical activity service providers for veterans with disabilities. Disability & Health Journal, 14(3), 101094. 10.1016/j.dhjo.2021.101094 33811009

[bibr10-00084174231160954] Farrance C. Tsofliou F. Clark C. (2016). Adherence to community based group exercise interventions for older people: a mixed-methods systematic review. Preventive Medicine, 87, 155–166. 10.1016/j.ypmed.2016.02.037 26921655

[bibr11-00084174231160954] Fong A. J. Saxton H. R. Kauffeldt K. D. Sabiston C. M. Tomasone J. R. (2021). “We’re all in the same boat together”: exploring quality participation strategies in dragon boat teams for breast cancer survivors. Disability & Rehabilitation, 43(21), 3078–3089. 10.1080/09638288.2020.1733676 32126196

[bibr12-00084174231160954] Harden S. M. McEwan D. Sylvester B. D. Kaulius M. Ruissen G. Burke S. M. Estabrooks P. A. Beauchamp M. R. (2015). Understanding for whom, under what conditions, and how group-based physical activity interventions are successful: a realist review. BMC Public Health, 15(1), 958. 10.1186/s12889-015-2270-8 26404722 PMC4582831

[bibr13-00084174231160954] Hsieh H.-F. Shannon S. E. (2005). Three approaches to qualitative content analysis. Qualitative Health Research, 15(9), 1277–1288. 10.1177/1049732305276687 16204405

[bibr14-00084174231160954] Jackson S. A. Ford S. K. Kimiecik J. C. Marsh H. W. (1998). Psychological correlates of flow in sport. Journal of Sport and Exercise Psychology, 20(4), 358–378. 10.1123/jsep.20.4.358

[bibr15-00084174231160954] Javorina D. Shirazipour C. H. Allan V. Latimer-Cheung A. E. (2020). The impact of social relationships on initiation in adapted physical activity for individuals with acquired disabilities. Psychology of Sport and Exercise, 50, 101752. 10.1016/j.psychsport.2020.101752

[bibr16-00084174231160954] Jesus T. S. Bhattacharjya S. Papadimitriou C. Bogdanova Y. Bentley J. Arango-Lasprilla J. C. Kamalakannan S. , & The Refugee Empowerment Task Force, I. N. G. of the A. C. of R. M. (2021). Lockdown-related disparities experienced by people with disabilities during the first wave of the COVID-19 pandemic: scoping review with thematic analysis. International Journal of Environmental Research and Public Health, 18(12), 6178. 10.3390/ijerph18126178 34200979 PMC8228347

[bibr17-00084174231160954] Koch L. C. Sweet S. N. Man K. E. Arbour-Nicitopoulos K. P. Orr K. Bundon A. Latimer-Cheung A. E. Tomasone J. R. (2022). Exploring the relationship between quality and quantity of physical activity participation in community-based exercise programs for persons with physical disabilities. Adapted Physical Activity Quarterly, 1(aop), 1–19. 10.1123/apaq.2021-0168 35453125

[bibr18-00084174231160954] Lanzino D. Sander E. Mansch B. Jones A. Gill M. Hollman J. (2016). Life space assessment in spinal cord injury. Topics in Spinal Cord Injury Rehabilitation, 22(3), 173–182. 10.1310/sci2203-173 29339859 PMC4981012

[bibr19-00084174231160954] Lund M. L. Nygard L. (2004). Occupational life in the home environment: The experiences of people with disabilities. Canadian Journal of Occupational Therapy, 71(4), 243–252. 10.1177/000841740407100419 15586857

[bibr20-00084174231160954] Ma J. K. Martin Ginis K. A. (2018). A meta-analysis of physical activity interventions in people with physical disabilities: Content, characteristics, and effects on behaviour. Psychology of Sport and Exercise, 37, 262–273. 10.1016/j.psychsport.2018.01.006

[bibr21-00084174231160954] Martin J. J. (2013). Benefits and barriers to physical activity for individuals with disabilities: a social-relational model of disability perspective. Disability and Rehabilitation, 35(24), 2030–2037. 10.3109/09638288.2013.802377 23781907

[bibr22-00084174231160954] Martin Ginis K. A. Evans M. B. Mortenson W. B. Noreau L. (2017). Broadening the conceptualization of participation of persons with physical disabilities: a configurative review and recommendations. Archives of Physical Medicine and Rehabilitation, 98(2), 395–402. 10.1016/j.apmr.2016.04.017 27216222

[bibr23-00084174231160954] Martin Ginis K. A. van der Ploeg H. P. Foster C. Lai B. McBride C. B. Ng K. Pratt M. Shirazipour C. H. Smith B. Vásquez P. M. Heath G. W. (2021). Participation of people living with disabilities in physical activity: a global perspective. The Lancet, 398(10298), 443–455. 10.1016/S0140-6736(21)01164-8 34302764

[bibr33-00084174231160954] Ministry of Health. (2022). *Physical activity-Province of British Columbia*. Province of British Columbia. https://www2.gov.bc.ca/gov/content/health/managing-your-health/physical-activity

[bibr24-00084174231160954] Morgan W. J. (2010). What, exactly, is occupational satisfaction? Journal of Occupational Science, 17(4), 216–223. 10.1080/14427591.2010.9686698

[bibr25-00084174231160954] Mukaka M. M. (2012). A guide to appropriate use of correlation coefficient in medical research. Malawi Medical Journal, 24(3), 69–71. https://pubmed.ncbi.nlm.nih.gov/23638278 23638278 PMC3576830

[bibr26-00084174231160954] Neupert S. D. Lachman M. E. Whitbourne S. B. (2009). Exercise self-efficacy and control beliefs predict exercise behavior after an exercise intervention for older adults. Journal of Aging and Physical Activity, 17(1), 1–16. 10.1123/japa.17.1.1 19299835 PMC3740728

[bibr27-00084174231160954] Nowell L. S. Norris J. M. White D. E. Moules N. J. (2017). Thematic analysis: striving to meet the trustworthiness criteria. International Journal of Qualitative Methods, 16(1), 1609406917733847. 10.1177/1609406917733847

[bibr28-00084174231160954] O'brien N. Heaven B. Teal G. Evans E. H. Cleland C. Moffatt S. Sniehotta F. F. White M. Mathers J. C. Moynihan P. (2016). Integrating evidence from systematic reviews, qualitative research, and expert knowledge using co-design techniques to develop a web-based intervention for people in the retirement transition. Journal of Medical Internet Research, 18(8), e210. https://10.0.8.148/jmir.5790 27489143 10.2196/jmir.5790 PMC4989122

[bibr29-00084174231160954] O'cathain A. Murphy E. Nicholl J. (2008). The quality of mixed methods studies in health services research. Journal of Health Services Research and Policy, 13(2), 92–98. 10.1258/jhsrp.2007.007074 18416914

[bibr30-00084174231160954] Parasuraman A. Colby C. L. (2015). An updated and streamlined technology readiness Index: TRI 2.0. Journal of Service Research, 18(1), 59–74. 10.1177/1094670514539730

[bibr31-00084174231160954] Parker K. Uddin R. Ridgers N. D. Brown H. Veitch J. Salmon J. Timperio A. Sahlqvist S. Cassar S. Toffoletti K. Maddison R. Arundell L. (2021). The use of digital platforms for adults’ and adolescents’ physical activity during the COVID-19 pandemic (our life at home): survey study. Journal of Medical Internet Research, 23(2), e23389. 10.2196/23389 33481759 PMC7857525

[bibr32-00084174231160954] Petersen J. A. Naish C. Ghoneim D. Cabaj J. L. Doyle-Baker P. K. McCormack G. R. (2021). Impact of the COVID-19 pandemic on physical activity and sedentary behaviour: a qualitative study in a Canadian city. International Journal of Environmental Research and Public Health, 18(9), 4441. 10.3390/ijerph18094441 33922094 PMC8122654

[bibr34-00084174231160954] Rantanen T. Eronen J. Kauppinen M. Kokko K. Sanaslahti S. Kajan N. Portegijs E. (2021). Life-space mobility and active aging as factors underlying quality of life among older people before and during COVID-19 lockdown in Finland: a longitudinal study. The Journals of Gerontology. Series A, Biological Sciences and Medical Sciences, 76(3), e60–e67. 10.1093/gerona/glaa274 33125043 PMC7665359

[bibr35-00084174231160954] Reid H. Miller W. C. Esfandiari E. Mohammadi S. Rash I. Tao G. Simpson E. Leong K. Matharu P. Sakakibara B. Schmidt J. Jarus T. Forwell S. Borisoff J. Backman C. Alic A. Brooks E. Chan J. Flockhart E. Mortenson W. B. (2021). The impact of COVID-19-related restrictions on social and daily activities of parents, people with disabilities, and older adults: protocol for a longitudinal, mixed methods study. JMIR Research Protocols, 10(9), e28337. 10.2196/28337 34292163 PMC8412136

[bibr36-00084174231160954] Richardson E. V. Smith B. Papathomas A. (2017). Disability and the gym: experiences, barriers and facilitators of gym use for individuals with physical disabilities. Disability and Rehabilitation, 39(19), 1950–1957. 10.1080/09638288.2016.1213893 27626359

[bibr37-00084174231160954] Rimmer J. H. Riley B. Wang E. Rauworth A. Jurkowski J. (2004). Physical activity participation among persons with disabilities: Barriers and facilitators. American Journal of Preventive Medicine, 26(5), 419–425. 10.1016/j.amepre.2004.02.002 15165658

[bibr38-00084174231160954] Ritchie H. Mathieu E. Rodés-Guirao L. Appel C. Giattino C. Ortiz-Ospina E. Hasell J. Macdonald B. Beltekian D. Roser M. (2020). Coronavirus pandemic (COVID-19). *Our World in Data*. https://ourworldindata.org/covid-stay-home-restrictions. 10.1038/s41597-020-00688-8 PMC7545176 33033256

[bibr39-00084174231160954] Roberts M. K. Ehde D. M. Herring T. E. Alschuler K. N. (2021). Public health adherence and information-seeking for people with chronic conditions during the early phase of the COVID-19 pandemic. PM&R, 13(11), 1249–1260. 10.1002/pmrj.12668 34218517 PMC8441730

[bibr40-00084174231160954] Roohafza H. R. Afshar H. Keshteli A. H. Mohammadi N. Feizi A. Taslimi M. Adibi P. (2014). What’s the role of perceived social support and coping styles in depression and anxiety? Journal of Research in Medical Sciences : The Official Journal of Isfahan University of Medical Sciences, 19(10), 944–949. https://pubmed.ncbi.nlm.nih.gov/25538777 25538777 PMC4274570

[bibr41-00084174231160954] Saebu M. (2010). Physical disability and physical activity: a review of the literature on correlates and associations. European Journal of Adapted Physical Activity, 3(2), 37–55. 10.5507/euj.2010.008

[bibr42-00084174231160954] Santino N. Arbour-Nicitopoulos K. P. Sharma R. Graham J. D. Bassett-Gunter R. L. (2021). Physical activity and loneliness among adolescents with disabilities: examining the quality of physical activity experiences as a possible moderator. Disability and Health Journal, 14(3), 101060. 10.1016/j.dhjo.2021.101060 33478910

[bibr43-00084174231160954] Saraiva M. D. Apolinario D. Avelino-Silva T. J. De Assis Moura Tavares C. Gattás-Vernaglia I. F. Marques Fernandes C. Rabelo L. M. Tavares Fernandes Yamaguti S. Karnakis T. Kalil-Filho R. Jacob-Filho W. Romero Aliberti M. J. (2021). The impact of frailty on the relationship between life-space mobility and quality of life in older adults during the COVID-19 pandemic. The Journal of Nutrition, Health & Aging, 25(4), 440–447. 10.1007/s12603-020-1532-z PMC7678592 33786560

[bibr44-00084174231160954] Shirazipour C. H. Evans M. B. Leo J. Lithopoulos A. Martin Ginis K. A. Latimer-Cheung A. E. (2020). Program conditions that foster quality physical activity participation experiences for people with a physical disability: a systematic review. Disability and Rehabilitation, 42(2), 147–155. 10.1080/09638288.2018.1494215 30324815

[bibr45-00084174231160954] van der Ploeg H. P. van der Beek A. J. van der Woude L. H. V. van Mechelen W. (2004). Physical activity for people with a disability. Sports Medicine, 34(10), 639–649. 10.2165/00007256-200434100-00002 15335241

[bibr46-00084174231160954] Wolf S. Seiffer B. Zeibig J.-M. Welkerling J. Brokmeier L. Atrott B. Ehring T. Schuch F. B. (2021). Is physical activity associated with less depression and anxiety during the COVID-19 pandemic? A rapid systematic review. Sports Medicine, 51(8), 1771–1783. 10.1007/s40279-021-01468-z 33886101 PMC8060908

[bibr47-00084174231160954] Wolff D. Nee S. Hickey N. S. Marschollek M. (2021). Risk factors for Covid-19 severity and fatality: a structured literature review. Infection, 49(1), 15–28. 10.1007/s15010-020-01509-1 32860214 PMC7453858

[bibr48-00084174231160954] Zacher H. Rudolph C. W. (2021). Individual differences and changes in subjective wellbeing during the early stages of the COVID-19 pandemic. The American Psychologist, 76(1), 50–62. 10.1037/amp0000702 32700938

[bibr49-00084174231160954] Zigmond A. S. Snaith R. P. (1983). The hospital anxiety and depression scale. Acta Psychiatrica Scandinavica, 67(6), 361–370. 10.1111/j.1600-0447.1983.tb09716.x 6880820

[bibr50-00084174231160954] Zimet G. D. Dahlem N. W. Zimet S. G. Farley G. K. (1988). The multidimensional scale of perceived social support. Journal of Personality Assessment, 52(1), 30–41. 10.1207/s15327752jpa5201_2 2280326

