# Supplementary material for: Quality of Physical Activity Participation Among Adults with Disabilities Through Pandemic Restriction
Source: Can J Occup Ther. 2023 May 15;90(2):161–72. doi: 10.1177/00084174231160954 (PMC10189534; doi:10.1177/00084174231160954)
Supplement: sj-docx-1-cjo-10.1177_00084174231160954 - Supplemental material for Quality of Physical Activity Participation Among Adults with Disabilities Through Pandemic Restriction [file sj-docx-1-cjo-10.1177_00084174231160954.docx]

# **Supplemental 1. Interview Guide**

**Interview guide**

*Hello, my name is _____________ and I would like to start the interview by thanking you for your participation. It is greatly appreciated. The purpose of this interview is to explore and gain a deeper understanding of your experiences and perceptions during the COVID-19 pandemic.*

*Throughout the interview, you will be asked about your personal experiences. The interview will take approximately 60 minutes. Per the Informed Consent you signed, I just want to remind you that your participation is voluntary. You can stop the interview at any time, if you no longer want to participate. You also do not need to answer any questions which make you feel uncomfortable. During the interview, my main job is to listen to you and your stories. I look forward to hearing about your experiences.*

*As indicated in the Informed Consent, this session will be video recorded and then transcribed. I will let you know when I will start recording. If your name happens to come up in the interview, it will be removed when the interview is transcribed and replaced with a pseudonym. Do you have any questions for me before we start? I am now going to start recording.*

1. Tell me what you’ve learned over the past month during COVID.
2. What has your experience of COVID been like?

Prompt: Emotionally, physically (exercise), socially.

Prompt: Who, if anyone, do you discuss how you feel about COVID with?

1. What was a typical day like for you before the COVID quarantine?

Prompt: What does a typical day look like for you now?

Prompt: What is the most meaningful part of your day?

Prompt: Which of these changes do you feel are positive?

Prompt: Which of the changes do you feel are negative?

1. What are you doing more of?
2. What are you doing less of?
3. What activities are you doing to connect with others?
   Prompt: How would you describe your living situation?
4. What activities are you doing to contribute?
   Prompt: To your personal well-being, to society, to the world?
5. What type of restorative activities are you engaging in?
   Prompt: How do you feel when you are doing your daily activities?
6. How, if at all, has the meaning of the activities you do changes with the advent of COVID?
7. What are some of the biggest challenges you have encountered?
8. What are some of the strategies and supports you have used to overcome them?
9. Knowing the ways you’ve adjusted to the current situation, what would you recommend to others?
10. What ideas do you have about innovations that could facilitate that change?
    Prompt 1. How, if it all, has your use of social media changed during this time?
    Prompt 2: Describe your use of technology currently.
11. What are your future plans?

Prompt: Today, this week, monthly, before next interview, for the year

1. You will have the option to participate in a “photovoice” part of this study where you take photos of your experiences during quarantine and share them during the next interview, or send them to us online. You will also have the option of giving the research team consent for these photos to be used beyond the interview in the report and in subsequent publications and presentations. Taking and sharing photos is entirely voluntary, and the photos will only be used as a part of this project. Photos will be stored on an encrypted server. Please let us know whether or not you wish to participate in the photovoice in the survey we send to you before our next interview and sign the Photo Release Form if you choose to send and share photos with the research team.

If the “photovoice” is something you’d like to participate in, please feel free to start taking pictures of your experiences during the pandemic to share!

**At interview’s end:**

*I would like to thank you for taking the time to share your experiences. They will be helpful to our research. You will receive your payment as was mentioned previously. I also just want to remind you that if you’re agreeable to taking photos up until the next interview, please bring those to the next interview so we can discuss them.*
Inform participant of upcoming email contact for this next part, if participating.  *Thank you again. Have a great evening!* [turn off the recording]

**Subsequent interviews**

*Hi, again! I would like to start the interview by thanking you for your continuing participation. It is greatly appreciated.*

*Much like our last interview, you will be asked about your personal experiences. The interview will take approximately 60 minutes. Per the Informed Consent you signed, I just want to remind you that your participation is voluntary. You can stop the interview at any time, if you no longer want to participate. You also do not need to answer any questions which make you feel uncomfortable. During the interview, my main job is to listen to you and your stories. I look forward to hearing about how things have gone since we met!*

*As indicated in the Informed Consent, this session will be video recorded and then transcribed. I will let you know when I will start recording. If your name happens to come up in the interview, it will be removed when the interview is transcribed and replaced with a pseudonym. Do you have any questions for me before we start? I am now going to start recording.*

1. Since the last time we talked how have things been going for you?
2. Have you experienced any changes of (e.g., living situation, health, etc.)
3. What is a typical day like for you?
4. How, if at all, has your experience of COVID changed since we last talked?
5. How, if at all, has COVID affected what a typical day is like for you?
6. What activities are you doing to connect with others?
   Prompt: How would you describe your living situation?
7. What activities are you doing to contribute?
8. What type of restorative activities are you engaging in?
   Prompt: How do you feel when you are doing your daily activities?
9. How, if at all, has the meaning of the activities you do changes with the advent of COVID?
10. What are some of the biggest challenges you have encountered?
11. What are some of the strategies and supports you have used to overcome them?
12. What are some potential things that you would think would be helpful for people who are in similar situations as you?
13. What do you think should happen in that regard?
14. What ideas do you have about innovations that could facilitate that change?
    Prompt 1. How, if it all, has your use of social media changed during this time?
    Prompt 2: Describe your use of technology currently?
15. (If consented to photovoice) Please describe each picture you took
    Prompt: Why did you take this picture? What does it convey about your experience?
16. What are your future plans?
