# Supplementary material for: Quality of Physical Activity Participation Among Adults with Disabilities Through Pandemic Restriction
Source: Can J Occup Ther. 2023 May 15;90(2):161–72. doi: 10.1177/00084174231160954 (PMC10189534; doi:10.1177/00084174231160954)
Supplement: sj-docx-2-cjo-10.1177_00084174231160954 - Supplemental material for Quality of Physical Activity Participation Among Adults with Disabilities Through Pandemic Restriction [file sj-docx-2-cjo-10.1177_00084174231160954.docx]

# **Supplemental 2. Correlation Matrix**

| **Composite Correlation Matrix** | | | | | | | | | | | | | | | |
| --- | --- | --- | --- | --- | --- | --- | --- | --- | --- | --- | --- | --- | --- | --- | --- |
|  | | MeEAP Mean | MeEAP Autonomy | MeEAP Belongingness | MeEAP Challenge | MeEAP Engagement | MeEAP Mastery | MeEAP Meaning | Age | TRI-2 | HADS-Anxiety | HADS-Depression | CD-RISC-25 (Resilience) | MSPSS (Social Support) | LSA |
| Age | Pearson Correlation | 0.093 | 0.051 | 0.089 | 0.032 | 0.174 | -0.050 | 0.195 |  |  |  |  |  |  |  |
|  | Sig. (2-tailed) | 0.475 | 0.698 | 0.493 | 0.804 | 0.179 | 0.701 | 0.133 |  |  |  |  |  |  |  |
| TRI-2 | Pearson Correlation | 0.112 | -0.082 | 0.160 | 0.087 | 0.168 | -0.013 | .306^*^ | 0.051 |  |  |  |  |  |  |
|  | Sig. (2-tailed) | 0.388 | 0.531 | 0.219 | 0.505 | 0.195 | 0.923 | 0.016 | 0.698 |  |  |  |  |  |  |
| HADS-Anxiety | Pearson Correlation | -.292^*^ | -0.176 | -.349^**^ | -.360^**^ | -0.176 | -.262^*^ | -0.159 | -0.218 | -0.234 |  |  |  |  |  |
|  | Sig. (2-tailed) | 0.023 | 0.175 | 0.006 | 0.004 | 0.175 | 0.041 | 0.221 | 0.092 | 0.069 |  |  |  |  |  |
| HADS-Depression | Pearson Correlation | -0.055 | 0.072 | -0.119 | -0.159 | -0.039 | 0.001 | -0.062 | -0.122 | -0.159 | .785^**^ |  |  |  |  |
|  | Sig. (2-tailed) | 0.673 | 0.580 | 0.359 | 0.220 | 0.768 | 0.991 | 0.636 | 0.350 | 0.220 | 0.000 |  |  |  |  |
| CD-RISC-25 | Pearson Correlation | 0.222 | 0.066 | 0.245 | 0.175 | 0.176 | 0.174 | .330^**^ | 0.186 | .426^**^ | -.480^**^ | -.533^**^ |  |  |  |
|  | Sig. (2-tailed) | 0.086 | 0.613 | 0.057 | 0.176 | 0.175 | 0.180 | 0.009 | 0.151 | 0.001 | 0.000 | 0.000 |  |  |  |
| MSPSS (Social Support) | Pearson Correlation | 0.125 | -0.005 | .259^*^ | 0.136 | 0.053 | 0.100 | 0.110 | .292^*^ | 0.194 | -.360^**^ | -.315^*^ | .424^**^ |  |  |
|  | Sig. (2-tailed) | 0.337 | 0.969 | 0.044 | 0.296 | 0.683 | 0.443 | 0.400 | 0.022 | 0.134 | 0.004 | 0.013 | 0.001 |  |  |
| LSA | Pearson Correlation | 0.013 | 0.106 | -0.027 | 0.044 | -0.116 | 0.115 | -0.069 | 0.137 | -0.132 | -.253^*^ | -.259^*^ | 0.220 | 0.179 |  |
|  | Sig. (2-tailed) | 0.921 | 0.418 | 0.838 | 0.734 | 0.372 | 0.380 | 0.599 | 0.291 | 0.312 | 0.049 | 0.044 | 0.089 | 0.168 |  |
| **. Correlation is significant at the 0.01 level (2-tailed). | | | | | | | | | | | | | | | |
| *. Correlation is significant at the 0.05 level (2-tailed). | | | | | | | | | | | | | | |  |
| c. Listwise N=61 | | | | | | | | | | | | | | |  |
